# Effect of Massachusetts House Bill No. 4196 on electronic cigarette use: a mixed-methods study

**DOI:** 10.1186/s12954-021-00498-0

**Published:** 2021-05-05

**Authors:** Amanda Katchmar, Adrian Gunawan, Michael Siegel

**Affiliations:** 1 School of Public Health, Boston University, 715 Albany St, Boston, MA 02118 USA; 2 Boston University School of Medicine, 72 E Concord St, Boston, MA 02118 USA

**Keywords:** E-cigarettes, Smoking tobacco, Vaping, Policy, Taxation

## Abstract

**Background:**

Electronic cigarettes, or e-cigarettes, are devices that deliver nicotine-containing aerosol and were used by 2.8% of American adults in 2017. Many people who smoke cigarettes have used e-cigarettes for smoking cessation, and the general consensus among health providers is that while vaping is not harmless, it is less harmful than smoking. To try to reduce youth e-cigarette use, the Commonwealth of Massachusetts imposed a 75% excise tax on nicotine-containing vaping products and banned the sale of all flavored tobacco products, including combustible tobacco, effective June 1, 2020. This tax, like similar taxes in other states, aimed to reduce e-cigarette consumption. However, past research has found that e-cigarettes and cigarettes are economic substitutes, meaning that an increase in e-cigarettes prices may push more people who smoke e-cigarettes to smoke combustible cigarettes.

**Methods:**

To determine the impacts of several events, such as the e-cigarette and vaping-associated lung injury (EVALI) outbreak and implementation of the Massachusetts e-cigarette tax, on e-cigarette and cigarette purchasing, we conducted an interrupted time-series analysis of year-on-year consumer purchasing data to impute changes in e-cigarette and cigarette purchasing in the Greater Boston area and the entire USA after several intervention points. We then surveyed a subset of people who used e-cigarettes to evaluate the plausibility that some e-cigarette consumers would travel out-of-state to purchase e-cigarettes.

**Results:**

The purchasing data indicated that there was no significant decrease in e-cigarette purchases in the Greater Boston convenience market after tax implementation. However, we found that e-cigarette purchases decreased significantly while cigarette purchases increased after several bans on e-cigarettes and numerous policy statements related to the EVALI outbreak. The survey results suggested that people who smoke e-cigarettes did not decrease their consumption after the implementation of the tax, but instead obtained e-cigarettes outside of Massachusetts.

**Conclusion:**

These results suggest that the Massachusetts flavor ban and tax did not reduce e-cigarette consumption in the Greater Boston area, and that messaging questioning the safety of e-cigarettes led to an increase in combustible cigarette use. This suggests the need for health authorities to reconsider how they communicate the relative risks of smoking and vaping.

**Supplementary Information:**

The online version contains supplementary material available at 10.1186/s12954-021-00498-0.

## Background

Although the prevalence of cigarette smoking among adults in the USA fell to an all-time low of 13.7% in 2018, partly due to the emergence of electronic cigarettes, smoking remains the leading cause of preventable disease and death in the USA, accounting for disease in over 16 million Americans and 20% of annual deaths [1–3]. Thus, while clinicians, public health advocates, and policymakers have introduced initiatives that decreased smoking rates over the past several decades, some professionals are still concerned about tobacco consumption. The emergence of electronic cigarettes, battery-operated devices that heat and vaporize nicotine-containing solutions for inhalation and are sold in disposable and rechargeable varieties also known as e-cigarettes, over the past decade has led to debate among health professionals over the risks and benefits of e-cigarettes, their relationship to tobacco smoking, and the question of how e-cigarettes should be regulated [4].

In 2017, 2.8% of American adults used e-cigarettes [5]. Since e-cigarettes deliver nicotine without the thousands of toxicants present in tobacco smoke and simulate the act of smoking, they are widely regarded as safer alternatives to combustible cigarettes as well as potential smoking cessation aids and harm reduction devices [6]. Although neither the Food and Drug Administration (FDA) nor the United States Preventive Services Task Force (USPSTF) have approved e-cigarettes for smoking cessation, many people who smoke have nevertheless utilized e-cigarettes in their attempts to stop smoking, and some clinicians see value in this approach [4, 7]. In 2013, 56% of Montana adults who used e-cigarettes reported doing so to “[try] to quit or reduce cigarette use,” and a survey of North Carolina physicians in the same year indicated that 67% of those surveyed thought that e-cigarettes would aid in stopping smoking, with 35% recommending e-cigarettes to their patients [8, 9]. On a national scale, a CDC (US Centers for Disease Control and Prevention) study of smoking cessation methods used by adults who smoked between 2014 and 2016 found that 35.3% of participants who used multiple methods during their most recent cessation attempt replaced some cigarette intake with e-cigarettes, and 24.7% switched completely from cigarettes to e-cigarettes [10].

Despite the general consensus that e-cigarettes are less harmful than combustible cigarettes, opponents of harm reduction have maintained that they are not harmless, and that in addition to containing nicotine, which can be dependency-forming, e-cigarette aerosols have been found to include particulates, flavorings and volatile organic compounds, carcinogens, and heavy metals like nickel, tin, and lead [4, 6]. The CDC has alarmed the public by reporting that e-cigarettes have increased in popularity among teenagers and young adults to become the most commonly used tobacco product among youth, with 7.6% of high school students and 0.9% of middle school students reporting “frequent” e-cigarette use in 2020 [4, 11]. This has led some physicians to be reluctant to recommend e-cigarettes to their smoking patients, despite evidence of their effectiveness in promoting smoking cessation in a randomized clinical trial [12].

Adding to the concerns of e-cigarette critics, 2019 saw a nationwide outbreak of what the CDC mistakenly called “e-cigarette or vaping product use-associated lung injury,” or “EVALI,” that sickened over 2800 people (many of whom were under the age of 34) and killed 68 by February 2020 [13, 14]. Although it was later found that the cause of EVALI was vitamin E acetate, found in vaporizable THC products and not e-cigarettes, many governments and health organizations utilized the outbreak to restrict e-cigarette use [15].

The outbreak prompted Governor Charlie Baker of Massachusetts to declare a public health emergency and announce a 3-month ban on the sale of all vaping products in Massachusetts on September 24, 2019 [14]. On November 27, 2019, Governor Baker signed Massachusetts House Bill No. 4196 (“An Act Modernizing Tobacco Control”) into law, imposing a 75% excise tax on nicotine-containing vaping products and banning the sale of all flavored tobacco products, including menthol cigarettes and e-cigarettes, except in licensed smoking bars beginning on June 1, 2020 [16]. In Massachusetts, smoking bars are defined as establishments in which the primary activity is the sale of tobacco products for on-site consumption, and there are only 27 permitted establishments in the state. Thus, they are unlikely to be substitutes for other establishments where e-cigarettes can be purchased [17, 18].

While House Bill No. 4196 made Massachusetts the first state in the country to permanently ban retail sales of all flavored tobacco products, Massachusetts is neither the first nor the only state to tax e-cigarettes, which are taxed in 22 other states and the District of Columbia [19, 20]. Additionally, on December 20th, 2019, a new federal law was enacted that raised the minimum age to purchase any form of tobacco to 21 years of age [21]. Given that taxes on combustible tobacco products have proven to be potent deterrents of combustible cigarette use, it is unsurprising that many state policymakers have turned to taxation and regulation to deter youth e-cigarette use and raise revenue for governments [5, 22–24]. However, many experts worry that high taxes and restrictive regulations on e-cigarettes lead to an increase in cigarette smoking, whose destructive health effects are well-established, and it is thus important for policymakers to strike an appropriate balance between deterring both youth e-cigarette use and adult combustible cigarette use when regulating e-cigarettes [6, 24].

In principle, taxation reduces consumption of a product by rendering it more expensive. The sensitivity of consumer demand for a product to changes in price is known as the *price elasticity of demand* (PED), which denotes the percent change in the consumption of a product in response to a 1% increase in price [22]. Studies from the International Agency for Research on Cancer, Community Preventive Services Task Force, and National Cancer Institute in collaboration with the World Health Organization estimate the PED of combustible cigarettes to be approximately − 0.4, implying that a 10% rise in cigarette prices reduces cigarette purchasing by 4% [22, 25–27]. Given the growing significance of e-cigarettes in the tobacco market, several studies have sought to quantify the PED for these products, including four that analyzed Nielsen retail scanner data to calculate PED values for e-cigarettes ranging from − 1.2 to − 2.054 [28–31]. In addition, Pesko and Warman [32] employed Nielsen data to investigate e-cigarette PED specifically among youth and reported a 3.3% decrease in youth e-cigarette use with a $1 price increase, and Pesko et al. [33] later estimated that the number of days middle- and high-school students used e-cigarettes fell by 9.7% with a 10% price increase. However, Nielsen [34] retail scanner data do not include sales from vaping shops and online sources, which constitute 45% of all US vaping-related sales; utilizing an experimental auction, Corrigan et al. calculated a PED of − 0.56 for e-cigarettes, which is consistent with the PED for combustible cigarettes [27].

While the aforementioned results indicate that e-cigarettes are a prime tax target to deter vaping among youth, it is important for policymakers to consider the *cross-price elasticity of demand* of combustible cigarettes with respect to e-cigarette prices, or the percent change in combustible cigarette consumption in response to a 1% increase in e-cigarette prices. A negative cross PED indicates that two products are complements, whereas a positive PED indicates that they are substitutes. Some studies have found a complementary relationship between e-cigarettes and combustible cigarettes; when Cotti et al. examined data from the Nielsen Consumer Panel to determine whether tobacco control policies such as taxes and clean-air laws affected e-cigarette consumption, they discovered that households were 22% less likely to purchase e-cigarettes with the implementation of a $1 excise tax on cigarettes, and Abouk and Adams found that bans on e-cigarette sales to minors (effectively equivalent to infinite price increases) reduced youth cigarette smoking by 15% [35, 36]. However, because the latter study did not measure the effects of e-cigarette bans on individual consumers, it cannot be determined whether the reduction in youth cigarette use was attributable to the decision of potential new cigarette consumers to not smoke or to the discontinuation of tobacco use by past consumers.

Still, other studies suggest that the two products are economic substitutes, supporting the view that excessively taxing e-cigarettes may drive some e-cigarette consumers to smoke cigarettes and even blunt the effectiveness of existing cigarette taxes [28, 31–33, 37–39]. For instance, in a discrete choice experiment, Pesko et al. found that increasing e-cigarette prices from $3 to $6 reduced the likelihood of selecting e-cigarettes over combustible cigarettes by 13.6%, while Cotti et al. used Nielsen retail scanner data spanning 2011 to 2017 to measure the effect of e-cigarette taxes in eight states and concluded that 6.4 additional combustible cigarette packs were purchased for every disposable e-cigarette pod not purchased due to an e-cigarette tax [28, 40]. Studying the effects of Minnesota’s 95% excise tax on vaping products, which took effect on July 1, 2015, Saffer et al. calculated that a 10% rise in e-cigarette prices prompted a 13% increase in cigarette consumption and concluded that the tax increased adult smoking rates and reduced quit rates by 1.14%, estimating that 32,400 additional adults who smoked cigarettes would have stopped smoking in the absence of the tax [20, 38]. Pesko and Warman also studied the effect of the Minnesota tax on youth e-cigarette use and found that higher e-cigarette taxes decreased youth consumption of e-cigarettes, along with evidence of cross-price elasticity between e-cigarettes and combustible cigarettes; taken together, this suggests that e-cigarette taxes may lead to an increase in smoking [32]. More recently, Yang et al. used a convenience sample to study the effect of San Francisco’s ban on flavored tobacco products on young adults and similarly found that banning flavored products led to a decrease in flavored tobacco use and an increase (though not statistically significant) in cigarette use. Several participants in the authors’ survey indicated that they were able to avoid complying with the ban by “stocking up” before the ban went into effect, purchasing products outside of the city, or making illicit purchases within the city [41].

In light of Saffer et al.’s evaluation of Minnesota’s e-cigarette tax and the recent imposition of a similar tax in Massachusetts, the objective of our study was to assess the short-term effects of MA House Bill No. 4196 on combustible and electronic cigarette consumption among Massachusetts adults who smoke e-cigarettes. In addition, we aimed to evaluate the impact of EVALI, the state ban on the sale of electronic cigarettes, the removal of that ban, and the COVID-19-related shutdown on both cigarette and e-cigarette consumption in Massachusetts compared to the USA as a whole. Since Massachusetts is geographically small and surrounded by four states with no or lower e-cigarette taxes, it was hypothesized that the tax would not reduce e-cigarette use among adults in the Greater Boston area and would instead drive them to purchase vaping products outside the state [20]. We also hypothesized that the EVALI scare would sharply reduce electronic cigarette consumption, but at the expense of an increase in the consumption of combustible cigarettes.

To test these hypotheses, we utilized Nielsen data on cigarette and electronic cigarette sales in Massachusetts and the USA as a whole to directly measure any changes in purchasing after messaging and policy changes related to the EVALI outbreak, the subsequent ban on e-cigarettes in Massachusetts, the implementation of COVID-19 emergency measures, and the implementation of the Massachusetts excise tax through an interrupted time series analysis of year over year data with a control group. In an ancillary analysis, we utilized online surveys administered approximately 20–30 days before and after the tax commencement date of June 1, 2020, to generate hypotheses that might help us interpret the results from the analysis of consumer purchasing data. This study contributes valuable new knowledge to the public health literature not only because it is the first study of its kind to evaluate Massachusetts’s 75% excise tax on e-cigarettes, but also because it is the first to empirically analyze the impact of the EVALI scare on both electronic cigarette and tobacco cigarette consumption.

## Methods

### Design overview

We conducted a two-part study to assess the combined impacts of several events, including the EVALI outbreak, Massachusetts excise e-cigarette tax, and flavored tobacco product ban.

*Part 1* Nielsen ScanTrack data, which track the UPC information of products sold at partnering retailers around the country, were utilized to measure changes in the purchasing behavior of Greater Boston area e-cigarette consumers, and US consumers at large. The dataset we analyzed contained information about the Tobacco Alternatives (Vapor) and Cigarette categories, which refer to UPC-coded electronic and combustible cigarettes, sold in the Greater Boston area and US convenience channels; the dataset contained data for 136 single weeks ending on September 5, 2020. The data were aggregate and not linked to any individual consumer or establishment. We tabulated the number of units and total sales of e-cigarettes and combustible cigarettes purchased in the Greater Boston area and the USA as a whole before and after several intervention cut points and then determined whether there was any change in consumer purchasing habits after each point by conducting an interrupted time-series analysis using Stata Statistical Software, Release 16 [42].

*Part 2* A survey was designed to explore the possibility that individuals would obtain e-cigarettes outside of the Commonwealth of Massachusetts to evade the excise tax and flavor ban. Prior to the implementation of the excise tax, a baseline survey (see Additional File 1) of 36 adults who consumed e-cigarettes was conducted between May 3 and May 31, 2020 to measure the frequency of e-cigarette use among participants and assess the type(s) of e-cigarettes they used; where they obtained devices and cartridges; and whether they also smoked combustible cigarettes, marijuana, or marijuana-based vaporizable products. In the month following the implementation of the tax, participants were asked to complete a follow-up survey (see Additional File 2) containing the same questions along with questions related to their knowledge of the tax. The data were then analyzed to assess whether the excise tax had a measurable effect on participants’ use of e-cigarettes and/or other types of legal combustible/vaporizable drugs.

### Part 1

#### Data source and measures

The Nielsen ScanTrack dataset we analyzed included the number of units sold for each product in the Tobacco Alternatives (Vapor) and Cigarette categories, along with the average price per unit sold. The dataset included information regarding 16,416,873,473 e-cigarette and combustible cigarette purchases made between June 1, 2018 and September 5, 2020 at convenience stores in the Greater Boston area (Eastern Massachusetts, Southern New Hampshire, Rhode Island, Windham County in Vermont, and Windham County in Connecticut) and all purchases made at partnering retailers in the USA during the study period, encompassing an estimated 55% of all e-cigarette sales made during this time period in the Greater Boston area and the USA, respectively [34, 43]. The data were received as weekly aggregates and did not include any information about consumer characteristics, such as age or income, or any store-level data indicating the specific states where purchases were made. To facilitate comparison between sales in the Greater Boston area and sales in the USA, we divided all of the weekly sales figures in each respective group by the number of individuals in the two populations who were above the age of 16. Therefore, our outcomes of interest were the number of units purchased per capita and the per capita sales of cigarettes and e-cigarettes in the Greater Boston area and the USA before and after the implementation of the excise tax.

#### Interventions

We specified five different intervention cut points that correspond to: (1) the San Francisco ban on e-cigarettes and American Lung Association (ALA) statement on e-cigarettes from the week ending on June 29, 2019, which was closely followed by the EVALI outbreak; (2 and 3) the beginning and end of the Massachusetts ban on all vaping-related products that went into effect on September 24, 2019; (4) the beginning of COVID-19-related lockdown measures during the week of March 15, 2020; and (5) the implementation of the Massachusetts excise tax on e-cigarettes that went into effect on June 1, 2020.

- *Cut point 1* The interventions represented by this cut point are three-fold. First, on June 23, 2019, the American Lung Association issued a statement warning against the use of e-cigarettes [44], which was followed by similar warnings from other organizations. Then, on June 29, 2019, the San Francisco Board of Supervisors enacted a law banning the sale of e-cigarettes in the city [45], inspiring similar restrictions in other municipalities. In addition to these events, the EVALI outbreak began during August 2019 [46]. Our assessment of the changes in cigarette and e-cigarette purchasing trends during this intervention period therefore reflect the combined impact of the warning statements from several health organizations, e-cigarette ban in San Francisco, and the EVALI outbreak and scare.
- *Cut points 2 and 3* These cut points represent the week in which a ban on the sale of all vaping-related products went into effect in Massachusetts, and the week in which it was lifted [14]. The ban lasted until December 11, 2019 [47].
- *Cut point 4* This cut point represents the week in which many COVID-19 mitigation efforts were implemented, in both Massachusetts and across the USA [48].
- *Cut point 5* This cut point represents the week in which the excise tax on e-cigarettes and ban on flavored tobacco products went into effect in Massachusetts [16].

#### Data analysis

The changes in the number of e-cigarettes and combustible cigarettes purchased and the changes in weekly sales figures were analyzed via a year over year, interrupted time series analysis in Stata, following the methods described by Linden [49]. The function was used to estimate the effect of each event, as it is presumed that they would act as an “interruption” of normal e-cigarette buying behavior, and generates Newey–West standard errors for least-squares regression coefficients [49]. The percent change in e-cigarette and cigarette purchases per capita between corresponding weeks was calculated such that the amount of product purchased in one week was compared to the amount purchased a year prior; this form of analysis allows for better identification of changes in purchasing trends and is the preferred mode of analysis in the tobacco industry. Two multiple group analyses were conducted, comparing the weekly total sales figures for e-cigarettes and combustible cigarettes in the Greater Boston area to the corresponding rates in the USA as a whole. We assessed the changes in e-cigarette and cigarette sales that resulted from several events: three that were policy-related, and two that were naturally occurring. We hypothesized that news coverage and e-cigarette bans preceding the EVALI outbreak, the subsequent ban on e-cigarettes in Massachusetts, the implementation of coronavirus stay-at-home orders, and the Massachusetts excise tax would all be reflected in the data. Our proposed model followed a standard interrupted time series based on segmented linear regression with dummy variables for each intervention specified above. Thus, our model was as follows:

$$y = Z \times M \times W \left[ {a + b\left( T \right) + c\left( \gamma \right) + d\left( \delta \right) + e\left( {\upvarepsilon } \right) + f\left( {\upzeta } \right) + g\left( \eta \right)} \right]$$

The term *T* equals time in weeks since the start of the study period, $\gamma$ indicates the state of the EVALI-related policies and statements (0 if before the start of the pandemic, 1 if after), $\delta$ indicates the implementation of the Massachusetts ban on vaping-related products (0 if before, 1 if after), *ε* indicates the end of the Massachusetts ban on vaping-related products (0 if before, 1 if after), *ζ* indicates the implementation of COVID restriction measures (0 if before, 1 if after), and *η* indicates the state of the Massachusetts flavor ban and excise tax (0 if before, 1 if after). We also included interaction terms to account for the month (*M*), week of the month (*W*), and study group (*Z*; the Greater Boston area or USA).

First, we calculated the percent changes in sales per capita for each category and then imported the dataset into Stata; we then declared it to be a time-series dataset using the tsset function. We then used the itsa function to compare the Massachusetts data for each product type to the corresponding United Stated data and analyzed the results returned by Stata. We also controlled for monthly effects in our regression and utilized a lag period of 1 week.

For the data, which includes regions of states other than Massachusetts, we acknowledged that the number of e-cigarettes sold in convenience stores in the region might not change, as individuals may have begun to purchase e-cigarettes in areas outside of Massachusetts but within the dataset after June 1. However, we also acknowledged that a decrease in e-cigarette sales and increase in combustible cigarette sales at convenience stores in the Greater Boston area may still occur, given the size of the Boston metropolitan population within the state of Massachusetts. For all analyses, we considered a p value of 0.05 to be significant.

#### Results

**Fig. 1:**
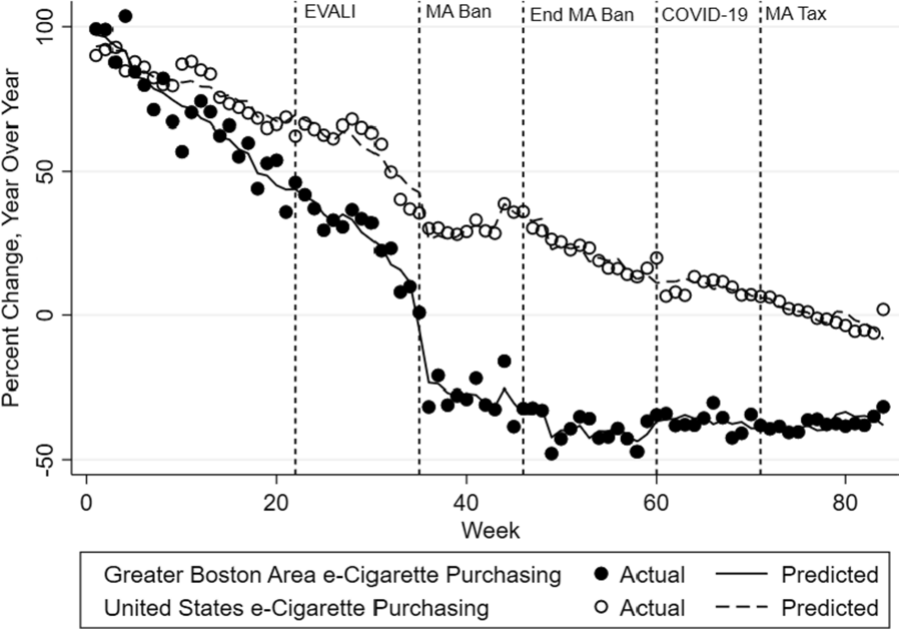
Interrupted time-series analysis of year-on-year e-cigarette sales per capita. This figure shows the data points and interrupted time-series regressions associated with each group. Vertical lines indicate the weeks in which the EVALI outbreak and related policy statements, beginning of the Massachusetts e-cigarette ban, end of the ban, coronavirus measures, and Massachusetts excise tax occurred, respectively

**Fig. 2:**
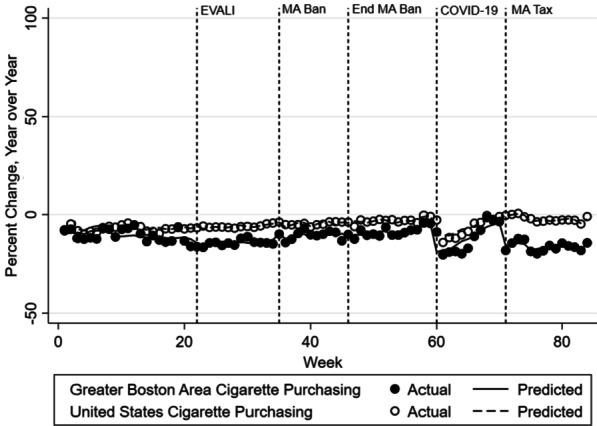
Interrupted time-series analysis of year-on-year cigarette sales per capita. This figure shows the data points and interrupted time-series regressions associated with each group. Vertical lines indicate the weeks in which the EVALI outbreak and related policy statements, beginning of the Massachusetts e-cigarette ban, end of the ban, coronavirus measures, and Massachusetts excise tax occurred, respectively

Our results for the regressions of e-cigarette and cigarette sales are summarized in Figs. 1 and 2, respectively. Figure 1 shows the trends for e-cigarette sales per capita in the Greater Boston area and the USA, with intervention cut points for the San Francisco ban, ALA press release, and EVALI outbreak; Massachusetts ban on e-cigarettes; end of the Massachusetts ban; COVID-19 mitigation measures; and Massachusetts excise tax, respectively. Prior to the first cut point, e-cigarette purchases in the Greater Boston area and the USA were decreasing relative to the prior year’s sales, with a smaller number of units purchased per capita in the Greater Boston area. After the passage of the e-cigarette ban in San Francisco and the statement from the ALA about the dangers of vaping, the e-cigarette purchases in both groups dropped, and purchases dropped even further after the Massachusetts ban on e-cigarettes, leveling off in the Greater Boston area and continuing to decrease in the USA after the ban was lifted. There was a slight increase in purchasing in the Greater Boston area after the coronavirus mitigation measures were implemented, and after the Massachusetts excise tax, the trend in purchasing stayed flat. In the USA, e-cigarette purchasing continued to decline compared to the previous year.

Figure 2 shows the trends for cigarette sales per capita in the Greater Boston area and the USA, with the same intervention time periods as Fig. 1. The trends for the Greater Boston area and the USA throughout the time period represented are mostly the same, with the USA having a larger number of sales per capita throughout the study period. Prior to the EVALI-related policy changes and statements, cigarette sales per capita were slightly increasing as compared to prior years in the USA as a whole, and there was a drop before a slight increase in sales in the Greater Boston area. After these events and subsequent ban on e-cigarettes in Massachusetts, both groups saw a steady increase in cigarette sales, staying steady after the ban was lifted. There was a steep drop in sales after COVID-19 safety measures were implemented, followed by a sharp increase in sales. Following the Massachusetts excise tax on e-cigarettes, there was a large drop in combustible cigarette sales in the Greater Boston area, which is not seen in the data for the USA.

We found that, prior to any intervention periods, the baseline rate of per capita e-cigarette unit purchasing in the USA as a whole was decreasing significantly by 1.30% from a base coefficient of 95.65 per week compared to the previous year (*p* value = 0.001, Table 1). The Greater Boston area rate was decreasing by a significantly greater amount compared to the USA, by 1.37% from a base coefficient of 4.14 per week (*p* value = 0.000, Table 1).

**Table 1:** E-cigarette regression coefficients, standard errors, *t* statistics, and *p* values

	Coefficient	Standard error	*t* statistic	*p* value
Baseline				
GBA				
Level	4.140104	3.051848	1.36	0.177
Trend	− 1.373532	0.2321417	− 5.92	0.000*
USA				
Level	95.65063	3.482517	27.47	0.000*
Trend	− 1.295596	0.3823137	− 3.39	0.001*
After EVALI				
GBA				
Level change	− 0.8664863	4.936645	− 0.18	0.861
Trend change	0.7472639	0.6716606	1.11	0.268
USA				
Level change	3.427075	4.571001	0.75	0.455
Trend change	− 1.770607	0.9661419	− 1.83	0.069
After mass ban				
GBA				
Level change	− 14.18386	6.428874	− 2.21	0.029*
Trend change	− 1.189679	0.941033	− 1.26	0.208
USA				
Level change	0.1329325	7.218978	0.02	0.985
Trend change	2.631157	1.278143	2.06	0.042*
After ban ended				
GBA				
Level change	− 0.0827453	4.759094	− 0.02	0.986
Trend change	2.808323	0.6655645	4.22	0.000*
USA				
Level change	− 3.341619	2.741816	− 1.22	0.225
Trend change	0.0170775	1.126806	0.02	0.988
After COVID− 19				
GBA				
Level change	6.271158	4.28192	1.46	0.145
Trend change	− 0.7918229	0.7104741	− 1.11	0.267
USA				
Level change	− 1.653717	3.959569	− 0.42	0.677
Trend change	− 0.3092617	0.8731348	− 0.35	0.724
After tax				
GBA				
Level change	0.5203217	3.957587	0.13	0.896
Trend change	0.9910441	0.7555539	1.31	0.192
USA				
Level change	2.519472	3.075552	0.82	0.414
Trend change	− 1.39879	0.8390774	− 1.67	0.098

Regression coefficients for the level and trend changes in e-cigarette purchasing in the Greater Boston area and the USA at the baseline and after the four intervention points, along with the standard error, *t* statistics, and *p* values corresponding to each value. Asterisks (*) indicate significance at* p* < 0.05

There was no significant change in the rate of decline in e-cigarette sales in the USA compared to the baseline trend during the period of the San Francisco ban and ALA statement about EVALI (p value for level change = 0.455, *p* value for trend change = 0.069, Table 1). Because the rate and level changes in the Greater Boston area were not significantly different than those of the USA, it also did not see a significant change in the rate of decline (*p* value for level change = 0.861, *p* value for trend change = 0.268, Table 1). Post-analysis results indicate that after EVALI, e-cigarette consumption was declining rapidly relative to the previous year in both the Greater Boston area and Massachusetts (GBA coefficient = − 3.6925, *p* value for coefficient = 0.0000; US coefficient = − 3.0662, *p* value for coefficient = 0.0003, Table 2). However, these rates of decline were not significantly different than each other (*p* value for difference = 0.3320, Table 2).

**Table 2:** E-cigarette post-trend estimated coefficients, standard errors, *t* values, *p* values, and confidence intervals

	Coefficient	Standard error	*t* value	*p* value	95% confidence interval	
Baseline						
GBA	4.140104	3.051848	1.36	0.177	− 1.898052	10.17826
USA	95.65063	3.482517	27.47	0.000*	88.76039	102.5409
EVALI						
GBA	− 3.6925	0.6550	− 5.6369	0.0000*	− 4.9885	− 2.3964
USA	− 3.0662	0.8282	− 3.7021	0.0003*	− 4.7049	− 1.4275
Difference	− 0.6263	0.6432	− 0.9737	0.3320	− 1.8988	0.6463
MA ban						
GBA	− 2.2510	0.7901	− 2.8490	0.0051*	− 3.8142	− 0.6878
USA	− 0.4350	0.7573	− 0.5745	0.5667	− 1.9334	1.0633
Difference	− 1.8159	0.6195	− 2.9312	0.0040*	− 3.0417	− 0.5902
After ban ended						
GBA	0.5744	0.6246	0.9196	0.3595	− 0.6614	1.8102
USA	− 0.4180	0.5692	− 0.7343	0.4641	− 1.5442	0.7082
Difference	0.9924	0.2911	3.4086	0.0009*	0.4164	1.5684
COVID-19						
GBA	− 0.5267	0.5933	− 0.8877	0.3763	− 1.7005	0.6471
USA	− 0.7272	0.6887	− 1.0559	0.2930	− 2.0899	0.6355
Difference	0.2006	0.6068	0.3305	0.7415	− 0.9999	1.4010
MA tax						
GBA	− 0.9344	0.4942	− 1.8907	0.0609	− 1.9122	0.0434
USA	− 2.1260	0.5320	− 3.9965	0.0001*	− 3.1785	− 1.0735
Difference	1.1916	0.4405	2.7051	0.0078*	0.3201	2.0631

The post-trend regression coefficients for e-cigarette purchasing in the Greater Boston area and the USA at baseline and after the four intervention points, the difference between the coefficients of the two groups, along with the standard error, *t* value, *p* statistic, and 95% confidence interval for each value. Asterisks (*) indicate significance at* p* < 0.05

During the week of the Massachusetts ban on e-cigarettes, the USA did not see an immediate change in the level of year over year purchasing compared to the previous period (*p* value for level change = 0.985, Table 1). However, the Greater Boston area saw a significant decrease in the level as compared to the USA (*p* value for level change = 0.029, Table 1). At this intervention point, the USA saw a significant increase in the trend of year over year purchasing compared to the previous period, becoming less negative (*p* value for trend change = 0.042, Table 1), and because the trend change for the Greater Boston area was not significantly different, it also saw a significant increase (*p* value for trend change = 0.208, Table 1). Post-analysis data indicate that e-cigarette consumption in the Greater Boston area was declining relative to the previous year, but the same did not hold true for the USA (GBA coefficient = − 2.2510, *p* value for coefficient = 0.0000; US coefficient = − 0.4350, *p* value for coefficient = 0.5667, Table 2). The trend in the Greater Boston area was significantly more negative than that in the USA (*p* value for difference = 0.0040, Table 2).

After the Massachusetts ban on e-cigarettes was lifted, the USA saw no significant changes compared to the previous period (*p* value for level change = 0.225, *p* value for trend change = 0.988, Table 1). However, while there was no significant change in the level of year over year purchasing in the Greater Boston area, there was an increase in the trend relative to the USA, meaning that e-cigarette consumption was declining less rapidly than during the ban (*p* value for level change = 0.986, *p* value for trend change = 0.000, Table 1). Post-analysis results indicate that trends in e-cigarette consumption were not significantly different than the previous year in both the Greater Boston area and the USA (GBA coefficient = − 0.5744, *p* value for coefficient = 0.3595; US coefficient = − 0.4180, *p* value for coefficient = 0.4641, Table 2). The decline in the Greater Boston area was significantly less negative than that in the USA (*p* value for difference = 0.0009, Table 2).

At the onset of COVID-19 mitigation measures, there were no significant changes in either the level or trend of e-cigarette purchasing in the USA compared to the previous period (*p* value for level change = 0.677, *p* value for trend change = 0.724, Table 1). Similarly, there were no changes in the Greater Boston area (*p* value for level change = 0.145, *p* value for trend change = 0.267, Table 1). Post-analysis data indicate that e-cigarette consumption continued to decline relative to the previous year in both the Greater Boston area and the USA (GBA coefficient = − 0.5267, *p* value for coefficient = 0.3763; US coefficient = − 0.7272, *p* value for coefficient = 0.2930, Table 2). There was no significant difference in the rates of decline of the Greater Boston area and the USA (*p* value for difference = 0.7415, Table 2).

After the implementation of the Massachusetts excise tax, there were no significant changes in the level or trend of e-cigarette purchasing in the USA compared to the previous period (*p* value for level change = 0.414, *p* value for trend change = 0.098, Table 1). The same holds true for the Greater Boston area (*p* value for level change = 0.896, *p* value for trend change = 0.192, Table 1). Post-trend analysis shows that after the tax, e-cigarette consumption continued to decline relative to the previous year in both the USA and the Greater Boston area (GBA coefficient = − 0.9344, *p* value for coefficient = 0.0609; US coefficient = − 2.1260, *p* value for coefficient = 0.0001, Table 2). The rate of decline in the Greater Boston area was significantly lower than that of the USA (*p* value for difference = 0.0078, Table 2).

Prior to any intervention periods, the baseline rate of per capita cigarette purchasing in the USA as a whole was significantly lower than the previous year (*p* value for level = 0.000, *p* value for trend change = 0.146, Table 3). The Greater Boston area purchasing rate was not significantly different than that of the USA (*p* value for level change = 0.498, *p* value for trend change = 0.146, Table 3).

**Table 3:** Combustible cigarette regression coefficients, standard errors, *t* statistics, and *p* values

	Coefficient	Standard error	*t* statistic	*p* value
Baseline				
GBA				
Level	− 1.260115	1.855424	− 0.68	0.498
Trend	− 0.2084171	0.1424893	− 1.46	0.146
USA				
Level	− 7.223334	1.684262	− 4.29	0.000*
Trend	− 0.1965167	0.1554116	− 1.26	0.208
After EVALI				
GBA				
Level change	− 3.081916	1.843995	− 1.67	0.097
Trend change	0.2512693	0.2184791	1.15	0.252
USA				
Level change	3.209894	1.221976	2.63	0.010*
Trend change	0.0570767	0.2734974	0.21	0.835
After mass ban				
GBA				
Level change	2.278667	1.824167	1.25	0.214
Trend change	− 0.0303285	0.2785707	− 0.11	0.913
USA				
Level change	1.288095	0.874842	1.47	0.143
Trend change	0.6289722	0.3434625	1.83	0.069
After ban ended				
GBA				
Level change	− 1.300539	1.978937	− 0.66	0.512
Trend change	0.1918814	0.237574	0.81	0.421
USA				
Level change	− 1.455614	1.241793	− 1.17	0.243
Trend change	− 0.3225095	0.4276565	− 0.75	0.452
After COVID-19				
GBA				
Level change	− 4.319313	4.899213	− 0.88	0.380
Trend change	0.4127087	0.6795817	0.61	0.545
USA				
Level change	− 11.23393	3.299207	− 3.41	0.001*
Trend change	0.7737937	0.4751412	1.63	0.106
After tax				
GBA				
Level change	− 13.61722	3.55943	− 3.83	0.000*
Trend change	− 0.4348779	0.687841	− 0.63	0.528
USA				
Level change	− 0.5232736	1.81891	− 0.29	0.774
Trend change	− 1.124816	0.4704655	− 2.39	0.018*

Regression coefficients for the level and trend changes in cigarette purchasing in the Greater Boston area and the USA at the baseline and after the four intervention points, along with the standard error, *t* statistics, and *p* values corresponding to each value. Asterisks (*) indicate significance at* p* < 0.05

After the EVALI outbreak, there was an immediate increase in the level of cigarette purchasing, compared to the previous period (*p* value for level change = 0.010, Table 3). The level change for the Greater Boston area was not significantly different from the level change of the USA, meaning that it also increased (*p* value for level change = 0.097, Table 3). There were no significant changes in the trends of cigarette purchasing in either the USA or the Greater Boston area (US *p* value = 0.835, GBA *p* value = 0.252, Table 3). Post-analysis results indicate that cigarette consumption in the USA and the Greater Boston area were not significantly different than the consumption from the previous year, nor were they significantly different from each other (GBA coefficient = − 0.0966, *p* value for coefficient = 0.6915; US coefficient = − 0.1394, *p* value for coefficient = 0.4287; *p* value for difference = 0.7808, Table 4).

**Table 4:** Combustible cigarette post-trend estimated coefficients, standard errors, *t* values, *p* values, and confidence intervals

	Coefficient	Standard error	*t* value	*p* value	95% confidence interval	
Baseline						
GBA	− 1.26012	1.855424	− 0.68	0.498	− 4.93112	2.410887
USA	− 7.22333	1.684262	− 4.29	0.000*	− 10.5557	− 3.89098
EVALI						
GBA	− 0.0966	0.2429	− 0.3977	0.6915	− 0.5771	0.3839
USA	− 0.1394	0.1756	− 0.7940	0.4287	− 0.4869	0.2080
Difference	0.0429	0.1537	0.2789	0.7808	− 0.2612	0.3469
MA ban						
GBA	0.5021	0.3471	1.4463	0.1505	− 0.1848	1.1889
USA	0.4895	0.2764	1.7709	0.0789	− 0.0574	1.0365
Difference	0.0125	0.2311	0.0542	0.9569	− 0.4448	0.4698
After ban ended						
GBA	0.3714	0.2163	1.7175	0.0883	− 0.0564	0.7993
USA	0.1670	0.2188	0.7635	0.4466	− 0.2658	0.5998
Difference	0.2044	0.0991	2.0629	0.0411*	0.0084	0.4004
COVID-19						
GBA	1.5579	0.6017	2.5891	0.0107*	0.3674	2.7485
USA	0.9408	0.4394	2.1414	0.0341*	0.0715	1.8101
Difference	0.6171	0.6770	0.9116	0.3637	− 0.7223	1.9565
MA tax						
GBA	− 0.0018	0.1805	− 0.0098	0.9922	− 0.3589	0.3554
USA	− 0.1840	0.1669	− 1.1023	0.2724	− 0.5143	0.1463
Difference	0.1822	0.1300	1.4018	0.1634	− 0.0750	0.4394

The post-trend regression coefficients for cigarette purchasing in the Greater Boston area and the USA at baseline and after the four intervention points, the difference between the coefficients of the two groups, along with the standard error, *t* value, *p* statistic, and 95% confidence interval for each value. Asterisks (*) indicate significance at* p* < 0.05

After the e-cigarette ban in Massachusetts was implemented, there were no significant changes, compared to the previous period, in either the level or trend of cigarette purchasing in the USA (*p* value for level change = 0.143, *p* value for trend change = 0.069, Table 3). Likewise, there were no significant changes in the level or trend of cigarette purchasing in the Greater Boston area (*p* value for level change = 0.214, *p* value for trend change = 0.913, Table 3). Post-analysis results indicate that cigarette consumption in the USA and the Greater Boston area were not significantly different than the consumption from the previous year, nor were they significantly different from each other (GBA coefficient = 0.5021, *p* value for coefficient = 0.1505; US coefficient = 0.4895, *p* value for coefficient = 0.0789; *p* value for difference = 0.9569, Table 4).

Similarly, after the e-cigarette ban was lifted, there were no significant changes in either the level or trend of cigarette purchasing in the USA compared to the previous period (*p* value for level change = 0.243, *p* value for trend change = 0.452, Table 3). There were no significant changes in the level or trend of cigarette purchasing in the Greater Boston area (*p* value for level change = 0.512, *p* value for trend change = 0.421, Table 3). Post-analysis data indicate that after the ban ended, the year over year trend in cigarette consumption in the Greater Boston area was significantly higher than that of the USA (*p* value for difference = 0.0411, Table 4). However, neither were significantly different from the trends in cigarette consumption during the previous year (GBA coefficient = 0.3714, *p* value for coefficient = 0.0833; US coefficient = 0.1670, *p* value for coefficient = 0.4466, Table 4).

After the coronavirus outbreak, there was an immediate decline in cigarette purchasing in the USA, relative to the previous period (*p* value for level change = 0.001, Table 3). Because the level change for the Greater Boston area was not significantly different than that of the USA, it also saw a significant, immediate decrease (*p* value for level change = 0.380, Table 3). There were no significant changes in the trends of cigarette purchasing in either the USA or the Greater Boston area (US *p* value = 0.106, GBA *p* value  = 0.545, Table 3). Post-analysis results indicate that at this intervention point, the trends in cigarette consumption in the Greater Boston area and the USA increased relative to the prior year (GBA coefficient = 1.5579, *p* value for coefficient = 0.0107; US coefficient = 0.9408, *p* value for coefficient = 0.3637, Table 4). However, these trends were not significantly different from each other (*p* value for difference = 0.3637, Table 4).

After the implementation of the excise tax, there was no significant change in the level of cigarette purchasing, compared to the previous period, in the USA (*p* value for level change = 0.774, Table 3). However, the level change in the Greater Boston area was significantly more negative than that of the USA, signifying a decrease in cigarette purchasing compared to the previous period (*p* value for level change = 0.000, Table 3). There was a significant decrease in the trend of year over year cigarette purchasing in the USA at this time point, and because the trend change for the Greater Boston area was not significantly different than that of the USA, it also saw a significant decrease (US *p* value = 0.018, GBA *p* value = 0.528, Table 3). Post-analysis data indicate that neither the purchasing trends in the Greater Boston area or the USA were significantly different than those from the previous year, nor were they significantly different from each other (GBA coefficient = − 0.0018, *p* value for coefficient = 0.9922; US coefficient = − 0.1840, *p* value for coefficient = 0.2724; *p* value for difference = 0.1634, Table 4).

### Part 2

#### Sample recruitment

Survey participants were recruited electronically via Craigslist, vaping-related mailing lists, and social media posts. To target Massachusetts adults who smoke e-cigarettes, a recruitment advertisement was sent via email to the state mailing lists of several e-cigarette stores and consumer organizations, including CASAA (Consumers for Smoke-Free Alternatives Association), SFATA (Smoke-Free Alternatives Trade Association), Vape Daddy’s (located in Newton and Framingham, MA), The Vape Shop (located in Boston, MA), and Worcester Vapor (located in Worcester, MA). The recruitment messaging explained the purpose and format of the study and included a link to the exempt information script, indicating that the study had been declared exempt by the Boston University Medical Center Institutional Review Board. Viewers were directed to the survey if they consented to participate in the study.

On the first page of the survey, potential participants were screened to ensure that they were at least 18 years of age and resided in the state of Massachusetts at the time of the study. At the end of the survey, participants were asked to enter the last four digits of their phone number as a means to link their responses in the first and follow-up surveys. They were then redirected to a new web page and asked to enter their email address to receive the link to the follow-up survey. The email address collection mechanism was separate from the survey dataset, so email addresses could not be linked to participants’ responses. No personally identifying information was obtained, and the study was therefore declared to be exempt from full human subjects review by the Boston University Medical Center Institutional Review Board.

#### Survey measures

The surveys measured whether or not participants, who used e-cigarettes at the start of the study, continued to use e-cigarettes after the tax was implemented and whether the frequency of their use changed. They also measured the number of participants who used combustible cigarettes, marijuana, and/or vaporizable THC (tetrahydrocannabinol) products, along with the changes in consumption of those products across the study period. Additionally, participants were asked where they purchased e-cigarettes and what type of device they used. The survey also measured self-reported behavior changes as a result of the excise tax, such as increases in combustible cigarette use or decreases in e-cigarette use, and asked about self-identified demographic characteristics, such as gender, age, and ethnicity.

#### Results

Summary statistics are listed in Table 5. Of the responses received from the preliminary and follow-up surveys, thirty-six were eligible for analysis. Fifty-eight percent of respondents were male (*n* = 21), fifty-six percent (*n* = 20) were between the ages of 20 and 44, and the remaining forty-four percent (*n* = 16) were between the ages of 45 and 64 (Table 5). The majority of respondents identified as white (97%, *n* = 35, Table 1), and one respondent identified as Black/African-American (3%, *n* = 1, Table 5). A majority of respondents reported an annual household income greater than $50,000 (Table 5).

**Table 5:** Summary statistics (*n* = 36)

	*N*	Percentage
Gender		
Male	21	58.33
Female	15	41.67
Age		
18–24	0	0
25–44	20	55.56
45–64	16	44.44
65 and older	0	0
Race		
White	35	97.22
Black/African-American	1	2.78
Asian	0	0
American Indian or Alaska Native	0	0
Native Hawaiian or Pacific Islander	0	0
Hispanic descent (percentage of respondents)	3	8.33
Approximate annual household income		
$49,999 and under	7	19.44
$50,000–$99,999	14	38.89
$100,000 or more	12	33.33
Did not provide	3	8.33

Summary statistics of participants who completed both the baseline and follow-up surveys

Table 6 describes the change in e-cigarette, combustible cigarette, vaporizable THC, and marijuana usage before and after the implementation of the tax. There was no change in the number of respondents who reported daily electronic cigarette use before and after June 1, 2020. While the numbers of respondents who reported daily use for combustible cigarettes, vaporizable THC, and marijuana use increased, these differences were not large.

**Table 6:** Change in usage (*n* = 36)

	*N*	Percentage
Daily e-cigarette use		
Prior to June 1	28	77.78
After June 1	28	77.78
Change	0	0
Any combustible cigarette use		
Prior to June 1	3	8.33
After June 1	4	11.11
Change	1	33.33
Any vaporizable THC use		
Prior to June 1	6	16.67
After June 1	7	19.44
Change	1	16.67
Any combustible marijuana use		
Prior to June 1	6	16.67
After June 1	7	19.44
Change	1	16.67

Number of participants who reported any e-cigarette, combustible cigarette, vaporizable THC, or marijuana use before and after June 1 and the percentage of the sample to which this corresponds

Table 7 describes the change in purchase location of each product; there was an increase in the number of respondents who indicated that they made trips to other states primarily to purchase e-cigarettes after June 1, 2020. However, there were no similar increases in the number of respondents who traveled to other states primarily to purchase the other three products. There was no change in the type of business from which respondents purchased most of their e-cigarettes, save for the “other/unspecified,” which consisted of free-form responses. These free-form responses did not offer any codable location.

**Table 7:** Purchase location change

Out-of-state purchasing (*N* = 36)	*N*	Percentage	
Made trips primarily to purchase product before 6/1/2020	19	52.78	
Made trips primarily to purchase product after 6/1/2020	25	69.44	
Change	6	31.58	

Purchase location	Before 6/1/2020 (*N* = 36)	After 6/1/2020 (*N* = 36)	Change (%)
Specialty shop	26	23	− 11.54
Non-specialty shop	0	1	100.00
Online specialty	15	12	− 20.00
Online third party	1	2	100.00
Friend/acquaintance	0	4	400.00
Did not purchase in specified period	0	3	300.00
Other/unspecified	1	3	200.00

Number of participants who reported traveling out of state for the primary purpose of purchasing e-cigarettes before and after June 1, number of participants indicating where they purchased e-cigarettes before and after June 1, and the percentage change in these numbers. Participants were able to select more than one purchase location

## Discussion

In this study, we found that after the implementation of the Massachusetts excise tax on e-cigarettes, there was no significant decrease in e-cigarette purchasing in the Greater Boston convenience market area as compared to the prior year’s purchasing patterns. We also found that there was a decrease in cigarette purchasing relative to the previous period immediately after the implementation of the tax; it is presumed that this is due to the ban on flavored tobacco products, which was also implemented at the time of the ban. In an exploratory survey of adults who consumed e-cigarettes about their e-cigarette use and purchasing habits before and after the implementation of the excise tax, we found that there was no change in the number of individuals who reported daily e-cigarette, combustible cigarette, vaporizable THC, or marijuana use (Table 6). However, more respondents reported that they made trips to other states primarily for the purpose of purchasing e-cigarettes (Table 7). These responses are consistent with the consumer purchasing data and suggest that the tax did not deter respondents from purchasing e-cigarettes, but only served to push them to purchase products outside of Massachusetts.

There are some limitations to this study that should be considered when interpreting the results, chiefly, the small participant pool for the survey. The survey was designed to explore the plausibility that at least some consumers would obtain e-cigarettes outside of the Commonwealth of Massachusetts immediately after the implementation of the excise tax. The number of participants recruited was negatively impacted by the COVID-19 outbreak, and this pool is not generalizable to the broader population. There may also have been recall bias on the part of participants, which could have skewed survey results; further, questions asking participants about their purchasing behavior before and after the implementation of the sales tax were asked wholly within the second survey, which again could have led to recall bias.

There were also limitations to our quantitative analysis. While the Nielsen data capture a large amount of consumer purchasing in the Greater Boston convenience market, they do not include all of the purchases made in the area during the time period, including purchases made over the Internet or at specialty shops. This dataset also includes purchasing data from other states, such as Rhode Island and New Hampshire, which may potentially obscure changes in purchasing patterns; unfortunately, this was the most granular dataset available. This study also did not examine the effects of the excise tax on e-cigarette use by minors, as the Nielsen data do not allow one to determine the age of the purchaser. In addition, though we attempted to control for the effects of the COVID-19 pandemic within our study, purchasing behavior may differ during the pandemic, so the long-term effects of the intervention points studied may also differ.

Despite these limitations, the analysis provides preliminary evidence that the rate of e-cigarette purchasing decreased significantly compared to the previous year in the USA after the EVALI outbreak and implementation of related bans. The number of cigarettes purchased in the USA after this intervention period increased significantly compared to the prior period, as the decrease in the trend of in year-over-year cigarette sales became less negative, suggesting that news of the EVALI outbreak and related bans pushed those who consumed e-cigarettes to purchase combustible cigarettes. If this was truly the case, this phenomenon should be cause for reconsideration of public health messaging regarding e-cigarettes; while there is a nonzero risk associated with e-cigarette use, it pales in comparison to the well-documented risks associated with combustible cigarette use, and care should be taken to not influence e-cigarette consumers to return to combustible tobacco use.

The data suggest that the Massachusetts excise tax on e-cigarettes did not significantly alter e-cigarette purchasing in the Boston area, though news of the EVALI outbreak and subsequent ban on the sale of e-cigarettes did. However, the fact that our dataset included sales from the Boston metropolitan area, which includes parts of Rhode Island and New Hampshire, may obscure any changes in the state of Massachusetts. For example, a report from the Tax Foundation found that cigarette purchasing increased in the states surrounding Massachusetts as purchasing dropped within Massachusetts due to the ban on all flavored tobacco products (including flavored cigarettes) included in Massachusetts House Bill No. 4196 [50]. Given this information, it is likely that consumers also traveled outside of Massachusetts to avoid the excise tax on e-cigarettes.

Many of the concerns surrounding e-cigarette use focus on use in young adults, and taxation and bans on e-cigarettes have been suggested as interventions to reduce initiation and use of e-cigarettes in teenagers and young adults. However, a study by Jun and Kim concluded that implementation of a tax on e-cigarettes decreased initiation and use in adults aged 25 to 34, but not in adults aged 18 to 24, suggesting that interventions such as this one are not achieving their stated goals [51]. Previous research has suggested that increasing the price of e-cigarettes leads to a reduction in use among middle- and high-school students, but further research is needed in order to determine if this occurred following the implementation of the Massachusetts excise tax [33]. Despite these shortcomings, we found that the amount of e-cigarettes purchased and rate at which they were purchased in Massachusetts did not significantly change after the implementation of this tax, suggesting that the intervention did not reduce the use of e-cigarettes in Massachusetts.

## Conclusion

To measure the effects of Massachusetts House Bill No. 4196, which implemented a 75% excise tax on vaping-related products and banned flavored tobacco products, we analyzed changes in e-cigarette and combustible cigarette purchasing data after multiple local e-cigarette bans, press release from the ALA regarding risks associated with e-cigarette use, and the EVALI outbreak; the beginning and end of the Massachusetts ban on e-cigarettes; the implementation of COVID-19 mitigation measures; and the implementation of the Massachusetts excise tax. We then conducted an exploratory survey of adults who consumed e-cigarettes about changes in their e-cigarette consumption as a result of the implementation of the excise tax to determine the plausibility that they would travel out of state to avoid the tax.

The consumer purchasing data indicated that there was no significant decrease in the number of e-cigarette purchases in the Greater Boston convenience market after the implementation of the excise tax. These findings suggest that the actions taken by the Commonwealth of Massachusetts did not effectively reduce e-cigarette consumption, but may have instead encouraged individuals to seek products online or in states with lower tax rates on e-cigarettes.

In our analysis of consumer purchasing data, we found that e-cigarette purchases decreased significantly while cigarette purchases increased after local bans on e-cigarette use, including a ban in San Francisco, along with numerous press releases related to EVALI in August 2019. Because e-cigarettes are thought to be a less harmful alternative to combustible cigarettes, this reversal is troubling. If governments wish to reduce e-cigarette use in their populations, they should engage in evidence-based policies that work to lower, not increase, the risk that is incurred by these populations, and design messaging that accurately portrays risk associated with behaviors such as e-cigarette and combustible cigarette use. Overall, the results of this study demonstrate a clear need for policymakers and health officials to effectively balance the risks of e-cigarette use and combustible cigarette use in their policies and messaging.

## Supplementary Information

**Additional file 1**. Baseline Survey Instrument. This file contains the outline of the survey completed by participants before the implementation of the Massachusetts excise tax.

**Additional file 2**. Follow-up Survey Instrument. This file contains the outline of the survey completed by participants after the implementation of the Massachusetts excise tax.

## Data Availability

The survey data used in the current study are available from the corresponding author upon reasonable request. The consumer purchasing data that support the findings of this study are available from the Nielsen Company, LLC, (https://www.nielsen.com/eu/en/) but restrictions apply to the availability of these data, which were used under license for the current study and are not publicly available. Data are, however, available from the authors upon reasonable request and with permission of the Nielsen Company, LLC.

## Abbreviations

ALA: American Lung Association
CASAA: Consumers for Smoke-Free Alternatives Association
CDC: Centers for Disease Control and Prevention
EVALI: E-cigarette or vaping product use-associated lung injury
FDA: Food and Drug Administration
MA: Massachusetts
PED: Price elasticity of demand
SE: Standard error
SFATA: Smoke-Free Alternatives Trade Association
THC: Tetrahydrocannabinol
USPSTF: United States Preventive Services Task Force
