# Supplementary material for: Effect of Massachusetts House Bill No. 4196 on electronic cigarette use: a mixed-methods study
Source: Harm Reduct J. 2021 May 5;18:50. doi: 10.1186/s12954-021-00498-0 (PMC8097113; doi:10.1186/s12954-021-00498-0)
Supplement: Supplementary file 1 — Additional file 1. Baseline Survey Instrument. This file contains the outline of the survey completed by participants before the implementation of the Massachusetts excise tax. [file 12954_2021_498_MOESM1_ESM.docx]

Baseline Survey

Start of Block: Informed Consent

1 You are being asked to voluntarily participate in a research study. We are doing this study to determine if Massachusetts House Bill H.4196 has a measurable effect on the actions of current electronic cigarette users.  If you agree, we will ask you to answer a few questions to confirm you are eligible to participate. If you are eligible, you will be asked to complete a brief survey about your electronic cigarette use twice, once before June 1st, and once after.  Your eligibility will be confirmed each time you take the survey.  The survey should take no more than 15 minutes to complete each time.  You will be asked to provide us your email address in order to send you the links to the surveys; however, this email address will not be linked to survey results in any way and will be destroyed after sending you the survey links. If you agree, we will ask you to answer a few questions to confirm you are eligible to participate. If you are eligible you will be asked to complete a brief survey about your electronic cigarette use twice, once before June 1st, and once after We will store your information in ways we think are secure. We will store paper files in locked filing cabinets. We will store electronic files in computer systems with password protection and encryption. However, we cannot guarantee complete confidentiality.   If you have any questions, please contact Amanda Katchmar at via email at katchmar@bu.edu or via phone at (617) 286-2051, or Dr. Michael Siegel via email at mbsiegel@bu.edu or via phone at (617) 358-1347.

2 I voluntarily consent to participating in this study.

- Yes (1)
- No (2)

Skip To: End of Survey If I voluntarily consent to participating in this study. = No

End of Block: Informed Consent

Start of Block: Screening

3 What is your age

________________________________________________________________

Skip To: End of Survey If Condition: What is your age Is Less Than 18. Skip To: End of Survey.

Q18 Are you currently living in the state of Massachusetts?

- Yes (1)
- No (2)

Skip To: End of Survey If Are you currently living in the state of Massachusetts? = No

End of Block: Screening

Start of Block: Usage

5 Do you currently use electronic cigarettes (also known as e-cigarettes, vapes, etc.), or have you used an electronic cigarette in the past two weeks?

- Yes (1)
- No (2)

Skip To: End of Survey If Do you currently use electronic cigarettes (also known as e-cigarettes, vapes, etc.), or have you... = No

6 How often, on average, do you use electronic cigarettes?

- More than once a day (1)
- Daily (2)
- 4-6 times a week (3)
- 2-3 times a week (4)
- Once a week (5)
- Less than once a week (6)

7 When did you start using electronic cigarettes?

- Within the past week (1)
- Within the past month (2)
- Within the past 2-3 months (3)
- Within the past 4-6 months (4)
- Within the past year (5)
- Within the past two years (6)
- Longer than two years ago (7)

End of Block: Usage

Start of Block: Products

8 What brand(s) of electronic cigarette do you use on a regular basis?

- JUUL (1)
- blu (2)
- NJOY (3)
- Mig (4)
- Om (5)
- Vuse (6)
- Kangertech (7)
- Other (please describe) (8) ________________________________________________

9 Where did you obtain the product(s) that you are currently using?

- Purchased at a specialty shop (1)
- Purchased at a non-specialty shop (e.g. a convenience or drug store) (2)
- Purchased online from a specialty retailer (3)
- Purchased online from a third-party retailer (e.g. eBay) (4)
- From a friend/acquaintance (5)
- Other (please describe) (6) ________________________________________________

End of Block: Products

Start of Block: Other Drug Use

10 Aside from electronic cigarettes, do you use any of the following?

- Combustible nicotine products (cigarettes, cigars, etc.)? (1)
- Vaporizable THC products (2)
- Combustible THC products (3)

Display This Question:

If Aside from electronic cigarettes, do you use any of the following? = Combustible nicotine products (cigarettes, cigars, etc.)?

11 How often do you use combustible cigarettes?

- More than once a day (1)
- Once a day (2)
- 4-6 times a week (3)
- 2-3 times a week (4)
- Once a week (5)
- Less than once a week (6)

Display This Question:

If Aside from electronic cigarettes, do you use any of the following? = Vaporizable THC products

12 How often do you use vaporizable THC products?

- More than once a day (1)
- Once a day (2)
- 4-6 times a week (3)
- 2-3 times a week (4)
- Once a week (5)
- Less than once a week (6)

Display This Question:

If Aside from electronic cigarettes, do you use any of the following? = Combustible THC products

13 How often do you use combustible THC products?

- More than once a day (1)
- Once a day (2)
- 4-6 times a week (3)
- 2-3 times a week (4)
- Once a week (5)
- Less than once a week (6)

End of Block: Other Drug Use

Start of Block: Demographics

14 Choose one or more races that you consider yourself to be:

- White (1)
- Black or African American (2)
- American Indian or Alaska Native (3)
- Asian (4)
- Native Hawaiian or Pacific Islander (5)
- Other (6) ________________________________________________

15 Are you Spanish, Hispanic, or Latino?

- Spanish (1)
- Hispanic (2)
- Latino (3)
- N/A (4)

16 What is your gender identity?

- Male (1)
- Female (2)
- Non-binary (3)
- Not listed (4) ________________________________________________

End of Block: Demographics

Start of Block: Identifier

17 What are the last four digits of your phone number (this will *only* be used to link your responses from both surveys)?

________________________________________________________________

End of Block: Identifier
