# Supplementary material for: Effect of Massachusetts House Bill No. 4196 on electronic cigarette use: a mixed-methods study
Source: Harm Reduct J. 2021 May 5;18:50. doi: 10.1186/s12954-021-00498-0 (PMC8097113; doi:10.1186/s12954-021-00498-0)
Supplement: Supplementary file 2 — Additional file 2. Follow-up Survey Instrument. This file contains the outline of the survey completed by participants after the implementation of the Massachusetts excise tax. [file 12954_2021_498_MOESM2_ESM.docx]

Follow-Up Survey

Start of Block: Informed Consent

Q1 You are being asked to voluntarily participate in a research study. We are doing this study to determine if Massachusetts House Bill H.4196 has a measurable effect on the actions of current electronic cigarette users.  If you agree, we will ask you to answer a few questions to confirm you are eligible to participate. If you are eligible, you will be asked to complete a brief survey about your electronic cigarette use twice, once before June 1st, and once after.  Your eligibility will be confirmed each time you take the survey.  The survey should take no more than 15 minutes to complete each time.  You will be asked to provide us your email address in order to send you the links to the surveys; however, this email address will not be linked to survey results in any way and will be destroyed after sending you the survey links. If you agree, we will ask you to answer a few questions to confirm you are eligible to participate. If you are eligible you will be asked to complete a brief survey about your electronic cigarette use twice, once before June 1st, and once after We will store your information in ways we think are secure. We will store paper files in locked filing cabinets. We will store electronic files in computer systems with password protection and encryption. However, we cannot guarantee complete confidentiality.   If you have any questions, please contact Amanda Katchmar at via email at katchmar@bu.edu or via phone at (617) 286-2051, or Dr. Michael Siegel via email at mbsiegel@bu.edu or via phone at (617) 358-1347.

Q2 I voluntarily consent to participating in this study.

- Yes (1)
- No (2)

Skip To: End of Survey If I voluntarily consent to participating in this study. = No

End of Block: Informed Consent

Start of Block: Screening

Q3 What is your age

________________________________________________________________

Skip To: End of Survey If Condition: What is your age Is Less Than 18. Skip To: End of Survey.

Q4 Are you currently living in the state of Massachusetts?

- Yes (5)
- No (6)

End of Block: Screening

Start of Block: Usage

Q5 Do you currently use electronic cigarettes (also known as e-cigarettes, vapes, etc.), or have you used an electronic cigarette in the past two weeks?

- Yes (1)
- No (2)

Skip To: End of Survey If Do you currently use electronic cigarettes (also known as e-cigarettes, vapes, etc.), or have you... = No

Q6 How often, **since June 1, 2020,** did you use electronic cigarettes?

- More than once a day (1)
- Daily (2)
- 4-6 times a week (3)
- 2-3 times a week (4)
- Once a week (5)
- Less than once a week (6)

Q7 When did you start using electronic cigarettes?

- Within the past week (1)
- Within the past month (2)
- Within the past 2-3 months (3)
- Within the past 4-6 months (4)
- Within the past year (5)
- Within the past two years (6)
- Longer than two years ago (7)

Q8 **Since June 1, 2020,**has your electronic cigarette use increased, decreased, or stayed the same?

- Increased (1)
- Decreased (2)
- Stayed the same (3)
- Not sure (4)

End of Block: Usage

Start of Block: Products

Q9 What brand(s) of electronic cigarette **have you used since June 1, 2020?**

- JUUL (1)
- blu (2)
- NJOY (3)
- Mig (4)
- Om (5)
- Vuse (6)
- Kangertech (7)
- Other (please describe) (8) ________________________________________________

Q10 Where did you obtain the product(s) that you used **since June 1, 2020?**

- Purchased at a specialty shop (1)
- Purchased at a non-specialty shop (e.g. a convenience or drug store) (2)
- Purchased online from a specialty retailer (3)
- Purchased online from a third-party retailer (e.g. eBay) (4)
- From a friend/acquaintance (5)
- Other (please describe) (6) ________________________________________________

End of Block: Products

Start of Block: E-Cigarette Use Change

Q11 Which of the following BEST describes how you changed your e-cigarette use **after June 1, 2020?**

- I used e-cigarettes before June 1 but I no longer use them (1)
- I used e-cigarettes before June 1 and I still use the same amount (2)
- I used e-cigarettes before June 1 and I still use them, but less often (3)
- I used e-cigarettes before June 1 and I still use them, but more often (4)
- I did not use e-cigarettes before June 1 and I now use them (5)
- I did not use e-cigarettes before June 1 and I still do not use them (6)

Q12
Have you noticed any change in the amount of money you pay for e-cigarettes **since June 1, 2020**?

- Significantly more expensive (1)
- More expensive (6)
- No change (7)
- Cheaper (8)
- Significantly cheaper (9)

Q13 **Before June 1, 2020**, how often did you purchase e-cigarettes outside of Massachusetts?

- Never (1)
- Seldom (4)
- Sometimes (5)
- Fairly often (6)
- Often (7)

Display This Question:

If Before June 1, 2020, how often did you purchase e-cigarettes outside of Massachusetts? != Never

Q14 Where outside of Massachusetts did you purchase e-cigarettes?

________________________________________________________________

Q15 Have you been purchasing more e-cigarettes outside Massachusetts since June 1, 2020?

- Yes (1)
- No (2)

Display This Question:

If Have you been purchasing more e-cigarettes outside Massachusetts since June 1, 2020? = Yes

Q16 How much more?

- I used to buy most of my e-cigarettes in Massachusetts, but I now buy most of my e-cigarettes outside Massachusetts (1)
- Significantly more, but I still buy most of my e-cigarettes in Massachusetts (7)
- Somewhat more. but I still buy most of my e-cigarettes in Massachusetts (8)
- Slightly more, but I still buy most of my e-cigarettes in Massachusetts (9)
- I've always bought most of my e-cigarettes outside Massachusetts and am now buying even more e-cigarettes outside Massachusetts (10)

Display This Question:

If Have you been purchasing more e-cigarettes outside Massachusetts since June 1, 2020? = Yes

Q17 Why?

________________________________________________________________

________________________________________________________________

________________________________________________________________

________________________________________________________________

________________________________________________________________

Display This Question:

If If Why? Text Response Is Displayed

Q18 Where outside of Massachusetts have you been purchasing e-cigarettes?

________________________________________________________________

Q19 **Prior to June 1, 2020,** how often did you make trips to other states *primarily* for the purpose of purchasing e-cigarettes?

- Never (1)
- Seldom (4)
- Sometimes (5)
- Fairly often (6)
- Often (7)

Display This Question:

If Prior to June 1, 2020, how often did you make trips to other states primarily for the purpose of... != Never

Q20 Why?

________________________________________________________________

Q21 **Since June 1, 2020**, how often do you make trips to other states *primarily* for the purpose of purchasing e-cigarettes?

- Never (1)
- Seldom (4)
- Sometimes (5)
- Fairly often (6)
- Often (7)

Display This Question:

If Since June 1, 2020, how often do you make trips to other states primarily for the purpose of purc... != Never

Q22 Why?

________________________________________________________________

End of Block: E-Cigarette Use Change

Start of Block: Other Drug Use

Q23 Aside from electronic cigarettes, did you use any of the following**since June 1, 2020**?

- Combustible nicotine products (cigarettes, cigars, etc.)? (1)
- Vaporizable THC products (2)
- Combustible THC (marijuana) (3)

Q24 Which of the following BEST describes how you changed your combustible cigarette use **after June 1, 2020?**

- I used combustible cigarettes before June 1 but I no longer use them (1)
- I used combustible cigarettes before June 1 and I still use the same amount (2)
- I used combustible cigarettes before June 1 and I still use them, but less often (3)
- I used combustible cigarettes before June 1 and I still use them, but more often (4)
- I did not use combustible cigarettes before June 1 and I now use them (5)
- I did not use combustible cigarettes before June 1 and I still do not use them (6)

Q25 Which of the following BEST describes how you changed your vaporizable THC use **after June 1, 2020**?

- I used vaporizable THC before June 1 but I no longer use it (1)
- I used vaporizable THC before June 1 and I still use the same amount (2)
- I used vaporizable THC before June 1 and I still use it, but less often (3)
- I used vaporizable THC before June 1 and I still use it, but more often (4)
- I did not use vaporizable THC before June 1 and I now use it (5)
- I did not use vaporizable THC before June 1 and I still do not use it (6)

Q26 Which of the following BEST describes how you changed your combustible THC (marijuana) use **after June 1, 2020**?

- I used combustible THC (marijuana) before June 1 but I no longer use it (1)
- I used combustible THC (marijuana) before June 1 and I still use it the same amount (2)
- I used combustible THC (marijuana) before June 1 and I still use it, but less often (3)
- I used combustible THC (marijuana) before June 1 and I still use it, but more often (4)
- I did not use combustible THC (marijuana) before June 1 and I now use it (5)
- I did not use combustible THC (marijuana) before June 1 and I still do not use it (6)

End of Block: Other Drug Use

Start of Block: Cigarettes - Current

Q27 How often did you use combustible cigarettes **since June 1, 2020**?

- More than once a day (1)
- Once a day (2)
- 4-6 times a week (3)
- 2-3 times a week (4)
- Once a week (5)
- Less than once a week (6)

Q28 **Since June 1, 2020,**has your combustible cigarette use increased, decreased, or stayed the same?

- Increased (1)
- Decreased (2)
- Stayed the same (3)
- Not sure (4)

Q29
Have you noticed any change in the amount of money you pay for combustible cigarettes **since June 1, 2020**?

- Significantly more expensive (1)
- More expensive (6)
- No change (7)
- Cheaper (8)
- Significantly cheaper (9)

Q30 **Before June 1, 2020**, how often did you purchase combustible cigarettes outside of Massachusetts?

- Never (1)
- Seldom (4)
- Sometimes (5)
- Fairly often (6)
- Often (7)

Display This Question:

If Before June 1, 2020, how often did you purchase combustible cigarettes outside of Massachusetts? != Never

Q31 Where outside of Massachusetts did you purchase combustible cigarettes?

________________________________________________________________

Display This Question:

If Aside from electronic cigarettes, did you use any of the following since June 1, 2020? = Combustible nicotine products (cigarettes, cigars, etc.)?

Q32 Have you been purchasing more combustible cigarettes outside Massachusetts since June 1, 2020?

- Yes (1)
- No (2)

Display This Question:

If Have you been purchasing more combustible cigarettes outside Massachusetts since June 1, 2020?  = Yes

Q33 How much more?

- I used to buy most of my combustible cigarettes in Massachusetts, but I now buy most of my combustible cigarettes outside Massachusetts (1)
- Significantly more, but I still buy most of my combustible cigarettes in Massachusetts (7)
- Somewhat more. but I still buy most of my combustible cigarettes in Massachusetts (8)
- Slightly more, but I still buy most of my combustible cigarettes in Massachusetts (9)
- I've always bought most of my e-cigarettes outside Massachusetts and am now buying even more combustible cigarettes outside Massachusetts (10)

Display This Question:

If Have you been purchasing more combustible cigarettes outside Massachusetts since June 1, 2020?  = Yes

Q34 Why?

________________________________________________________________

________________________________________________________________

________________________________________________________________

________________________________________________________________

________________________________________________________________

Display This Question:

If If Text Response Is Displayed

Q35 Where outside of Massachusetts have you been purchasing combustible cigarettes?

________________________________________________________________

Q36 **Prior to June 1, 2020,** how often did you make trips to other states *primarily* for the purpose of purchasing combustible cigarettes?

- Never (1)
- Seldom (4)
- Sometimes (5)
- Fairly often (6)
- Often (7)

Display This Question:

If Prior to June 1, 2020, how often did you make trips to other states primarily for the purpose of... != Never

Q37 Why?

________________________________________________________________

Q38 **Since June 1, 2020**, how often do you make trips to other states *primarily* for the purpose of purchasing combustible cigarettes?

- Never (1)
- Seldom (4)
- Sometimes (5)
- Fairly often (6)
- Often (7)

Display This Question:

If Since June 1, 2020, how often do you make trips to other states primarily for the purpose of purc... != Never

Q39 Why?

________________________________________________________________

End of Block: Cigarettes - Current

Start of Block: Vaporizable THC - Current

Q40 How often did you use vaporizable THC products **since June 1, 2020**?

- More than once a day (1)
- Once a day (2)
- 4-6 times a week (3)
- 2-3 times a week (4)
- Once a week (5)
- Less than once a week (6)

Q41 **Since June 1, 2020**, has your vaporizable THC use increased, decreased, or stayed the same?

- Increased (1)
- Decreased (2)
- Stayed the same (3)
- Not sure (4)

Q42
Have you noticed any change in the amount of money you pay for vaporizable THC products **since June 1, 2020**?

- Significantly more expensive (1)
- More expensive (6)
- No change (7)
- Cheaper (8)
- Significantly cheaper (9)

Display This Question:

If Aside from electronic cigarettes, did you use any of the following since June 1, 2020? = Vaporizable THC products

Q43 Have you been purchasing more vaporizable THC products outside Massachusetts since **June 1, 2020**?

- Yes (1)
- No (2)

Display This Question:

If Have you been purchasing more vaporizable THC products outside Massachusetts since June 1, 2020?  = Yes

Q44 How much more?

- I used to buy most of my vaporizable THC products in Massachusetts, but I now buy most of my vaporizable THC products outside Massachusetts (1)
- Significantly more, but I still buy most of my vaporizable THC products in Massachusetts (7)
- Somewhat more. but I still buy most of my vaporizable THC products in Massachusetts (8)
- Slightly more, but I still buy most of my vaporizable THC products in Massachusetts (9)
- I've always bought most of my vaporizable THC products outside Massachusetts and am now buying even more vaporizable THC products outside Massachusetts (10)

Display This Question:

If Have you been purchasing more vaporizable THC products outside Massachusetts since June 1, 2020?  = Yes

Q45 Why?

________________________________________________________________

________________________________________________________________

________________________________________________________________

________________________________________________________________

________________________________________________________________

Display This Question:

If If Text Response Is Displayed

Q46 Where outside of Massachusetts have you been purchasing vaporizable THC products?

________________________________________________________________

Q47 **Prior to June 1, 2020,** how often did you make trips to other states *primarily* for the purpose of purchasing vaporizable THC products?

- Never (1)
- Seldom (4)
- Sometimes (5)
- Fairly often (6)
- Often (7)

Display This Question:

If Prior to June 1, 2020, how often did you make trips to other states primarily for the purpose of... != Never

Q48 Why?

________________________________________________________________

Q49 **Since June 1, 2020**, how often do you make trips to other states *primarily* for the purpose of purchasing vaporizable THC products?

- Never (1)
- Seldom (4)
- Sometimes (5)
- Fairly often (6)
- Often (7)

Display This Question:

If Since June 1, 2020, how often do you make trips to other states primarily for the purpose of purc... != Never

Q50 Why?

________________________________________________________________

End of Block: Vaporizable THC - Current

Start of Block: Combustible THC

Q51 How often did you use combustible THC (marijuana) **since June 1, 2020**?

- More than once a day (1)
- Once a day (2)
- 4-6 times a week (3)
- 2-3 times a week (4)
- Once a week (5)
- Less than once a week (6)

Q52 **In the past month**, has your combustible THC (marijuana) use increased, decreased, or stayed the same?

- Increased (1)
- Decreased (2)
- Stayed the same (3)
- Not sure (4)

Q53
Have you noticed any change in the amount of money you pay for combustible THC (marijuana) **since June 1, 2020**?

- Significantly more expensive (1)
- More expensive (6)
- No change (7)
- Cheaper (8)
- Significantly cheaper (9)

Q54 **Before June 1, 2020**, how often did you purchase combustible THC (marijuana) outside of Massachusetts?

- Never (1)
- Seldom (4)
- Sometimes (5)
- Fairly often (6)
- Often (7)

Display This Question:

If Before June 1, 2020, how often did you purchase combustible THC (marijuana) outside of Massachuse... != Never

Q55 Where outside of Massachusetts did you purchase combustible THC products?

________________________________________________________________

Q56 **Before June 1, 2020**, how often did you purchase combustible THC (marijuana) outside of Massachusetts?

- Never (1)
- Seldom (4)
- Sometimes (5)
- Fairly often (6)
- Often (7)

Display This Question:

If Before June 1, 2020, how often did you purchase combustible THC (marijuana) outside of Massachuse... != Never

Q57 Where outside of Massachusetts did you purchase combustible THC (marijuana)?

________________________________________________________________

Display This Question:

If Aside from electronic cigarettes, did you use any of the following since June 1, 2020? = Combustible THC (marijuana)

Q58 Have you been purchasing more combustible THC (marijuana) outside Massachusetts since **June 1, 2020**?

- Yes (1)
- No (2)

Display This Question:

If Have you been purchasing more combustible THC (marijuana) outside Massachusetts since June 1, 202... = Yes

Q59 How much more?

- I used to buy most of my combustible THC (marijuana) in Massachusetts, but I now buy most of my combustible THC (marijuana) outside Massachusetts (1)
- Significantly more, but I still buy most of my combustible THC (marijuana) in Massachusetts (7)
- Somewhat more. but I still buy most of my combustible THC (marijuana) in Massachusetts (8)
- Slightly more, but I still buy most of my combustible THC (marijuana) in Massachusetts (9)
- I've always bought most of my combustible THC (marijuana) outside Massachusetts and am now buying even more combustible THC (marijuana) outside Massachusetts (10)

Display This Question:

If Have you been purchasing more combustible THC (marijuana) outside Massachusetts since June 1, 202... = Yes

Q60 Why?

________________________________________________________________

________________________________________________________________

________________________________________________________________

________________________________________________________________

________________________________________________________________

Display This Question:

If If Text Response Is Displayed

Q61 Where outside of Massachusetts have you been purchasing combustible THC (marijuana)?

________________________________________________________________

Q62 **Prior to June 1, 2020,** how often did you make trips to other states *primarily* for the purpose of purchasing combustible THC (marijuana)?

- Never (1)
- Seldom (4)
- Sometimes (5)
- Fairly often (6)
- Often (7)

Display This Question:

If Prior to June 1, 2020, how often did you make trips to other states primarily for the purpose of... != Never

Q63 Why?

________________________________________________________________

Q64 **Since June 1, 2020**, how often do you make trips to other states *primarily* for the purpose of purchasing combustible THC (marijuana)?

- Never (1)
- Seldom (4)
- Sometimes (5)
- Fairly often (6)
- Often (7)

Display This Question:

If Since June 1, 2020, how often do you make trips to other states primarily for the purpose of purc... != Never

Q65 Why?

________________________________________________________________

End of Block: Combustible THC

Start of Block: H.4196

Q66 Are you aware of the 75% excise tax on electronic cigarette products that went into effect in Massachusetts on June 1, 2020?

- Yes (11)
- No (12)

End of Block: H.4196

Start of Block: Tax - Effects

Q67 In your opinion, has the tax influenced your purchasing and use of e-cigarettes?

- Yes, significantly (1)
- Yes, somewhat (2)
- No (3)

Q68 In your opinion, has the tax influenced your purchasing and use of combustible cigarettes?

- Yes, significantly (1)
- Yes, somewhat (2)
- No (3)

Q69 In your opinion, has the tax influenced your purchasing and use of vaporizable THC products?

- Yes, significantly (1)
- Yes, somewhat (2)
- No (3)

Q70 In your opinion, has the tax influenced your purchasing and use of combustible THC (marijuana)?

- Yes, significantly (1)
- Yes, somewhat (2)
- No (3)

Q71 Please indicate your attitude toward the new tax

- 1 - Strongly unfavorable (11)
- 2 (12)
- 3 - Somewhat unfavorable (13)
- 4 (14)
- 5 - Indifferent (15)
- 6 (16)
- 7 - Somewhat favorable (17)
- 8 (18)
- 9 - Strongly favorable (19)

Q72 Why?

________________________________________________________________

Q73 Do you believe that this tax will be beneficial to public health by deterring youths from trying vaping products?

- 1 - Strongly do not believe (20)
- 2 (21)
- 3 - Somewhat do not believe (22)
- 4 (23)
- 5 - Unsure (24)
- 6 (25)
- 7 - Somewhat believe (26)
- 8 (27)
- 9 - Strongly believe (28)

Q74 Why?

________________________________________________________________

End of Block: Tax - Effects

Start of Block: Demographics

Q75 Choose one or more races that you consider yourself to be:

- White (1)
- Black or African American (2)
- American Indian or Alaska Native (3)
- Asian (4)
- Native Hawaiian or Pacific Islander (5)
- Other (6) ________________________________________________

Q76 Are you of Hispanic descent?

- Yes (2)
- No (4)

Q77 What is your gender identity?

- Male (1)
- Female (2)
- Non-binary (3)
- Not listed (4) ________________________________________________

Q78 What is your approximate annual household income?

- Less than $10,000 (1)
- $10,000 to $19,999 (2)
- $20,000 to $29,999 (3)
- $30,000 to $39,999 (4)
- $40,000 to $49,999 (5)
- $50,000 to $59,999 (6)
- $60,000 to $69,999 (7)
- $70,000 to $79,999 (8)
- $80,000 to $89,999 (9)
- $90,000 to $99,999 (10)
- $100,000 to $149,999 (11)
- $150,000 or more (12)
- Prefer not to say (13)

End of Block: Demographics

Start of Block: Identifier

Q79 What are the last four digits of your phone number (this will *only* be used to link your responses from both surveys)?

________________________________________________________________

End of Block: Identifier
